# The Effect of Short and Long-Term Therapeutic Treatment with Insoles and Shoes on Pain, Function, and Plantar Load Parameters of Women with Plantar Fasciitis: A Randomized Controlled Trial

**DOI:** 10.3390/medicina58111546

**Published:** 2022-10-28

**Authors:** Ana Paula Ribeiro, Silvia Maria Amado João

**Affiliations:** 1 Biomechanics and Musculoskeletal Rehabilitation Laboratory, Health Science Post-Graduate Department, Medicine School, University Santo Amaro, São Paulo 04829-300, Brazil; 2 Physical Therapy Department, School of Medicine, University of São Paulo, São Paulo 01246-903, Brazil

**Keywords:** plantar fasciitis, treatment, foot, insoles, shoe, gait, pain, function

## Abstract

*Background and Objectives:* Plantar fasciitis (PF) is a prevalent musculoskeletal disease, with inflammation at the origin of the plantar fascia, that affects sedentary people, particularly middle-aged women. Foot pain and functional limitations lead patients to seek treatment. Investigate the therapeutic effect of conservative treatment combining a custom insole with minimalist flexible shoes and the shoes alone in a gait-training protocol, in the short and long term, in women with PF. *Materials and Methods:* Design: A randomized, controlled, and single-blind trial. Setting: Biomechanics laboratory. Participants: 36 women, 26 with acute PF and 10 controls. Intervention: Gait-training protocol wearing the minimalist shoes alone (SG, *n* = 12, age: 46.4 ± 9.6, height: 1.60 ± 0.2, BMI: 28.8 ± 4.2), with a custom insole in the shoes (CIG, *n* = 14, age: 48.9 ± 9.8, height: 1.60 ± 0.1, BMI: 26.7 ± 5.6), and control (CG, *n* = 10, age: 46.1 ± 10.7, height: 1.61 ± 0.2, BMI: 26.4 ± 4.8). Evaluations were performed at baseline (T0) and after three (T3) and six (T6) months. The intervention had a duration of six months (six hours a day, seven days a week). Primary outcomes were rearfoot pain (visual analogue scale), the Foot Function Index (FFI), Foot Health Status Questionnaire (FHSQ-Br), and 6 min walk test (6MWT). The secondary outcomes were plantar pressure distribution during gait, measured by the pressure platform, and foot posture. *Results:* The CIG was effective for reducing pain and improving the FPI after T6 compared to CG. The FPI, FHSQ-Br and 6MWT demonstrated improvements after T6 in both the CIG and SG, compared to the CG. After T6, contact area (rearfoot) and maximum force (forefoot) reduced with CIG. Maximum force (midfoot and rearfoot) reduced with CIG and SG, as did peak pressure (forefoot and midfoot) in relation to CG. *Conclusions:* A customized insole associated with minimalist flexible shoes during a gait-training protocol can be recommended as a more effective treatment than minimalist flexible shoes alone over the short and long term, for reduction in calcaneus pain, increased function and foot health, and improved walking through reduced plantar load in women with PF.

## 1. Introduction

Plantar fasciitis (PF) is a prevalent rheumatic musculoskeletal disease, with inflammation at the origin of the plantar fascia [1], which principally affects adults aged 50 years, particularly women [2] and athletes [3]. PF makes up approximately 25% of all foot injuries (acute and chronic stages) [4], and the cost of treatment ranges from $192 to $376 million [1]. A typical symptom of PF is pain in the heel, with consequences on walking, ascending and descending stairs, and individual autonomy [3,4,5].

Foot pain and functional limitations are dysfunctions of this disease that lead patients to seek and undergo some conservative treatments. Plantar overload, arch index, and rearfoot pronation have been strongly related to PF severity, i.e., fragmentation and degeneration of the collagen fibers of the plantar fascia [2,6,7,8,9,10,11,12,13,14], and its chronic progression (thickening of the plantar fascia with possible formation of bone spurs) [11]. Moreover, inadequate shoes, i.e., dense and rigid shoes that cause reductions in foot mobility or excessive pronation, are extrinsic biomechanical factors of the disease [2,15,16,17]. A reduction in the calcaneus overload, stretching the tension mechanism on the plantar fascia, is one of the main objectives of conservative treatment of PF [18]. However, the treatment is complex, due to poor understanding of the pathological mechanisms of PF, which begins with the acute phase, and progresses to the chronic phase (degeneration and/or calcaneal spurs) [3]. This progression includes remission periods and relapses, making it difficult for health professionals to direct the best therapeutic approach for PF [3,4,11,16].

Although the currently available pharmacotherapy with corticosteroid injections is sufficient to control pain [19,20], it is insufficient for the functional manifestations of the feet [21,22]. Furthermore, surgical treatment has been shown to reduce symptoms and increase foot function; however, it is related to high costs, prolonged leave from work and complications for normal foot functioning [23]. According to the literature, an increase in foot function and a reduction in biomechanical parameters aimed at plantar load produce positive effects [24,25,26,27,28]. Ankle night splints [15,16,29] and foot insoles are common short-term interventions for the management of PF [15,16]. Insoles with different designs tend to affect gait performance, i.e., heel cup and arch support are two important features for insole design [30]. The ergonomic heel cup design can reduce foot pressure in the heel area and an adequate arch support design can distribute plantar pressure evenly [30,31]. In addition, one study showed the feasibility and potential applications of a wearable insole pressure system for the ergonomic risk assessment of work-related musculoskeletal disorders [31]. The authors evaluated three insole types (general flat insole, ergo-insole, and insole padding system—IPS) for a period of 2 weeks during walking, and the results showed that the IPS was associated with lower plantar pressure on the midfoot and heel [31]. Using a single textured insole underfoot induced an asymmetry of anticipatory onsets in EMG activity in healthy subjects [32]. Another study showed that textured insole socks, used 3 days/week for 4 weeks, increased balance and decreased the risk of falls in older adults to the same extent as unmodified socks [33]. In other diseases, such as diabetes-related foot disorders, insoles of ethylene-vinyl acetate (EVA) were the most popular used by healthcare experts to reduce peak pressure on the forefoot by 37% during gait [34].

In this rationale, some studies have shown the therapeutic effects on gait of an insole (total contact with ethylene–vinyl acetate material) to reduce pain and plantar load on the calcaneus of patients with PF [35,36,37,38]. Despite this therapeutic efficacy, no studies in the literature with clinical trials, in the short (3 months) and long term (6 months), have compared the use of an insole with minimalist flexible shoes (without high heels) in patients with PF. The main shortcoming observed in the literature is that studies compare mechanical treatment with an insole with non-mechanical conservative treatments, such as stretching, physical therapy, and non-steroidal anti-inflammatory drugs [36]. Thus, it is necessary to understand and compare the therapeutic effect between two mechanical devices (insoles and shoes) on the pain and function of the feet of patients with PF, in order to aid in the development of a better strategy of mechanical treatment for this disease. 

The advantages of this type of mechanical treatment are its effectiveness in relieving the plantar overload on the feet, easy accessibility for the patient, and a lower chance of clinical complications compared to surgical treatment [3,10]. Evidence from the literature reveals that total contact of the insoles is more effective for relieving pain compared to heel cups. Other evidence reports the effectiveness of combining night splints or rocker shoes with insoles for pain relief and increased function [36], as well as the benefits of a clinical trial design with an insole, in the short and long term, combined with an exercise booklet [37] or shock therapy [38] to reduce pain and improve function of the feet [37,38]. However, no clinical studies have been performed in patients with PF using an insole combined with minimalist shoes (without high heels) in clinical trials with methodological rigor of blinding, randomization, and comparisons with a control.

More recently, the use of minimalist flexible shoes was shown to effectively reduce plantar overload, in the short and long term, in inflammatory diseases caused by impact forces [39,40,41,42,43,44]. The rationale for using this shoe is foot flexibility, which resulted in improved distribution of the plantar load and decreased impact forces on the knee [43,44]. The majority of scientific researchers believe that traditional high-heeled or rigid-soled shoes, frequently used by women, promote an increase in force on the knees and feet when walking, causing progressive disease [15,16]. Thus, it is extremely important to understand the therapeutic effect of the shoes, combined or not with the insole, in patients with PF, given the lack of evidence from clinical trials. If effective, the use of the minimalist shoe (inexpensive) might be part of a treatment prescription, representing a way to reduce costs associated with patient treatment. Our hypothesis was that the use of the minimalistic flexible shoes combined with the insole in women with PF, in the short and long term, could: (i) relieve rearfoot pain; (ii) increase foot function in daily living activities; and (iii) decrease plantar load during gait. Our aim was to investigate the therapeutic effect of conservative treatment combining a custom insole with minimalist flexible shoes and the shoes alone in a gait-training protocol, in the short and long term, in women with PF.

## 2. Materials and Methods

### 2.1. Study Design and Participant Recruitment

The design of the study was a randomized clinical trial with concealed allocation, blinding of assessors, and intention-to-treat analysis (six-month intervention) in women diagnosed with PF. The patients with PF allocated to the intervention groups received either the minimalist flexible shoes (SG) or custom orthopedic insoles combined with minimalist flexible shoes (CIG) on the first day and were required to wear them for three and six months consecutively, as the gait-training protocol. The patients allocated to the control group (CG) did not receive any intervention, only the standard protocol with guidelines on shoes for walking. The patients were assessed in the baseline condition (T0) and after three (T3) and six months (T6) after the intervention program.

**Figure 1:**
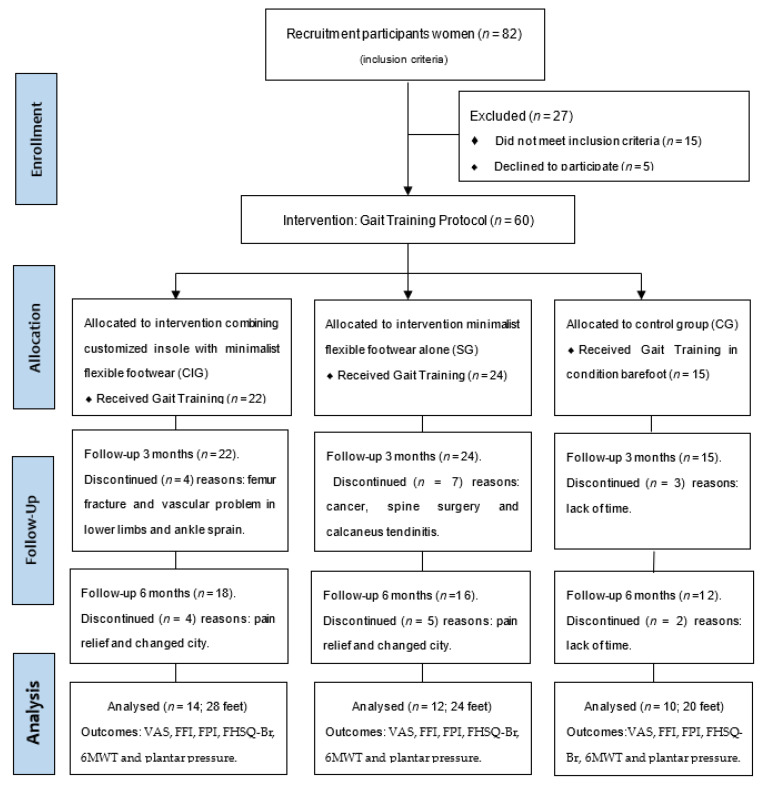
Flow Diagram of patients with plantar fasciitis to the clinical trial and measured outcomes.

The patients were recruited from two settings: (a) health center; (b) orthopedic and rheumatology clinics at the University Hospital. The diagnosis of PF was confirmed by a physical examination performed by an orthopedist and clinical exams: X-ray and ultrasonography (acute phase, without heel spurs) [3,44]. A total of 26 women with acute-phase PF were recruited and allocated to the intervention groups: SG (*n* = 12) and CIG (*n* = 14) and ten healthy women without PF to the CG (*n* = 10) (Figure 1).

The inclusion criteria were: female volunteers, aged between 30 and 55 years [2], body mass index (BMI) less than 35 kg/m^2^, diagnosis of PF, without a history of any surgical process in the knees, ankles, and hips or muscle injury in the previous six months, and without any diagnosed neurological and rheumatological disease [3,8,43], as well as rigid hallux, fasciotomy, calcaneal fractures, use of orthoses, arthroplasty (hip and knee), and corticosteroid injection in the heel. In addition, patients could not present ankle joint instability, mental disease or cognitive difficulty in answering information, and could not have used the minimalist shoe or similar for more than 24 h per week. During the study, concomitant treatments (physical therapy, shock waves, and/or acupuncture) were not allowed in order to avoid bias in interpreting the results [43,44]. 

The patients provided written consent, based on ethical approval from the Human Research Board of the local University (number: 1.074.141). Data access and storage comply with the National Health and Medical Research Council guidelines. This trial is registered in Clinical Trials (number NCT 03040557).

### 2.2. Randomization and Blinding

The randomization schedule was prepared using Clinstat software by an independent researcher who was not aware of the numeric code for the control and intervention groups. A numeric block randomization sequence was placed in opaque envelopes. After the patients’ agreement and assignment to participate in the research, the allocation into the groups was made by another independent researcher, who was also unaware of the codes. Only the physiotherapist responsible for the clinical trial knows who is receiving the intervention.

The clinical examinations were carried out by an orthopedist who was blind to the patient’s allocation. One physical therapist was responsible for pain, functional, and biomechanical assessments. Another physical therapist was responsible for follow-up and supervision of the intervention groups, providing instructions by telephone. Both physical therapists were blind to treatment allocation until the analysis had been completed [44].

### 2.3. Outcome Measures

The primary outcomes were calcaneus pain, measured by the Visual Analogue Scale (VAS), and feet functionality using the Foot Function Index (FFI), Foot Health Status Questionnaire (FSHQ-Br), and 6 min walk test (6MWT). The FFI evaluates the foot functional limitation. It contains 23 questions subdivided into three domains: foot pain, difficulty, and functional limitations. The final score varies from 0 to 100; the higher the score, the worse the functional condition [45]. The FHSQ-Br evaluates foot health. This questionnaire contains 29 questions, with a final score that varies from 0 to 100, representing from worse to better foot health [46]. The 6 min walk test (6MWT) was performed to evaluate functional capacity in walking. The women walked at maximum speed for 6 min along a 22 m track, and the total distance and number of turns were registered [47].

**Figure 2:**
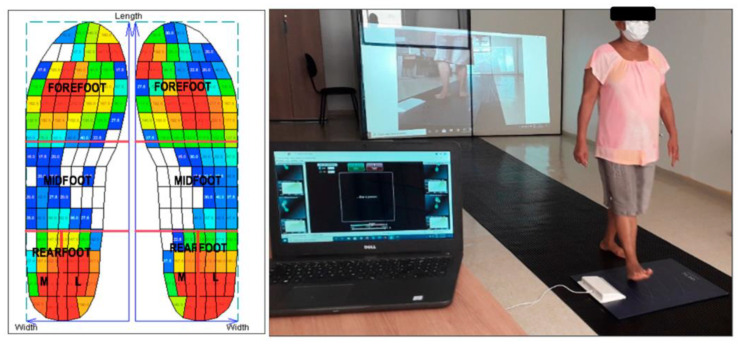
Analysis of plantar pressure distribution and division of foot areas.

The Secondary outcomes were plantar pressure distribution during walking using a pressure platform (Loran^®^ Sensor, LorAn Engineering Inc., Bologna, Italy), incorporating capacitance transducer sensors (4 sensors/cm^2^), and sampling at a frequency of 100 Hz. The patients walked freely along a 20 m walkway, with the pressure platform placed in the center of the walkway. The patients were asked to walk in a natural way (as performed in their daily lives) [43,44]. The cadence was monitored (by a metronome), but not controlled, ranging from 100 to 125 steps per minute [44]. After the adaptation of the patients, three attempts were allowed, and approximately 12 steps were acquired. The contact area (cm^2^), maximum force (N), and peak pressure (kPa) over the four plantar areas of the foot, medial and lateral rearfoot (30% of the foot length), midfoot (30% of the foot length), and forefoot and toes (40% of the foot length), were registered [32,33,34,35,36,37,38,39] (Figure 2). After this, the foot posture was verified by the FPI-6, to quantify the degree to which the foot could be supinated (−1 to −5), pronated (+6 to +10), or normal (0 to +5) [48]. 

### 2.4. Intervention Protocol

**Figure 3:**
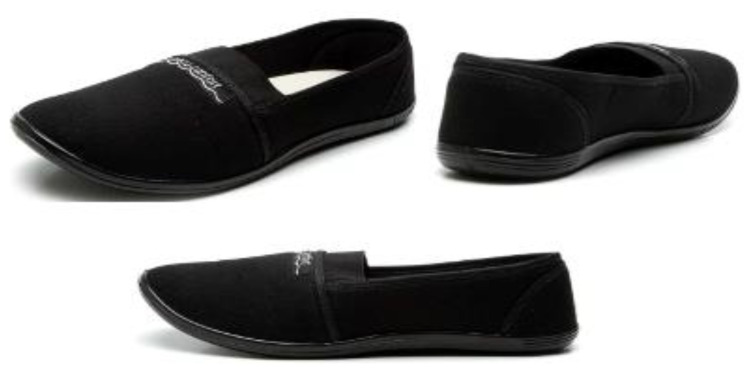
Minimalist flexible shoes worn for six months of intervention.

The gait-training program intervention was based on the daily use of the minimalist flexible shoe (SG) alone for three and six months, for six hours a day, seven days a week (at least 42 h per week). The minimalist flexible shoe (Shoes Moleca, Beira Rio S.A., Novo Hamburgo, RS, Brazil) used was a low-cost women’s double canvas, flexible, flat, walking shoe, without heel drop, with a 5 mm anti-slip rubber sole and a 3 mm internal wedge of ethylene vinyl acetate. The shoe has a mean weight of 0.172 ± 0.019 kg, with a mass of between 91 g and 182 g, depending on the shoes size [44] (Figure 3).

**Figure 4:**
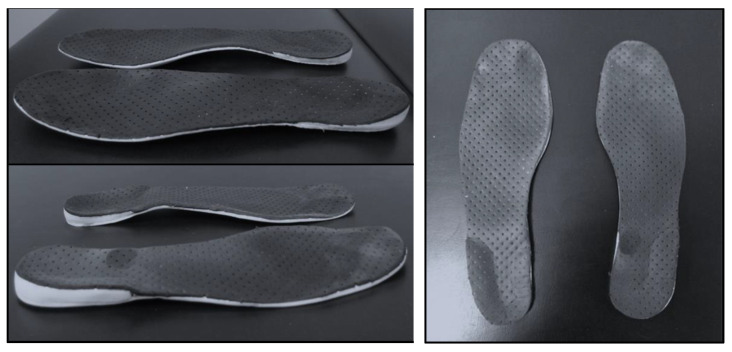
Custom orthopedic insole (total-contact with wedge at the side edge of the heel) combined with minimalist shoes for six months of intervention.

The patients allocated to the intervention received custom orthopedic insoles combined with the minimalist flexible shoe (CIG), also used daily. The customized insoles were flat, total-contact, weighed 31 g, were made of ethylene–vinyl acetate-EVA, were 3 mm thick in the forefoot with the support element under the plantar arch, and 7 mm in the rearfoot with the support element under the heel (wedge for the heel region), determining the two-point mechanical design. The insoles were manufactured for each patient individually, made with the patient standing on a foot foam mold, from which the EVA insole was fashioned. The gait-training protocol intervention was for three and six months, for six hours a day, seven days a week [44]. Every two weeks, the same physiotherapist phoned the patients in the intervention groups, SG or CIG, in order to verify adherence to the mechanical treatment and correct filling out of the diary. After three and six months of intervention, the shoes and insoles of the patients were photographed to verify the shoes were worn (Figure 4).

The patients allocated to the control group (CG) did not receive interventions, only the standard protocol, with guidelines on shoes for walking. During the intervention period, patients from the CG were asked not to wear minimalist flexible footwear or similar minimalist shoes and customized insoles, but they continued to receive their health care and painkiller medication at the hospital. At the end of the intervention period, all CG patients also received a free pair of minimalist flexible shoes with or without the customized insole.

### 2.5. Sample Size and Statistical Analysis

The sample size was performed considering calcaneus pain as the primary outcome, with an effect size of 0.30 (moderate). In total, 36 women were needed to provide an 80% power to detect a moderate effect difference between groups, considering a significance level of 0.05. The power of an experiment is the probability that the effect is detected. It is usually arbitrarily set to 0.8 or 0.9 (i.e., the investigator seeks an 80 or 90% chance of finding statistical significance if the specified effect exists). Note that power, symbolized as β, is the chance of obtaining a false-negative result (i.e., the experiment fails to reject an untrue null hypothesis or to detect the specified treatment effect).

The tests were based on intention-to-treat analysis, using the two-way ANOVA test to detect treatment–time and inter-group interactions (α = 5%). The effect size, using Cohen’s D, was measured between T0 and after six months (T6) of intervention, considering values of 0.20–0.39 as small, 0.40–0.79 as medium, and >0.80 as large effect sizes.

## 3. Results

Overall, 60 women initially demonstrated interest in participating (Figure 1), among which 36 participants were enrolled from February 2016 to December 2019. Characteristics of the groups were similar at baseline and after three and six months of intervention (Table 1).

### 3.1. Primary Outcomes

The interventions with SG or CIG promoted a reduction in calcaneus pain and functionality in the women with PF. However, the insole (CIG) was more effective in reducing pain when compared to the shoe alone (SG), after three and six months, with a medium effect size. Improved foot function was also positive with CIG and SG after three and six months. Considering the inter-group assessments, the CIG was better at increasing foot function when compared to the SG and CG after six months of treatment. Another important finding was the decrease in FPI after three and six months with both the SG and CIG (Table 2). 

Considering the FSHG-Br, a significant increase was observed after six months with both the insole (CIG) and shoe (SG); however, a medium effect size was observed for the CIG and small for the SG. Inter-group, both the insole and shoe showed improvement only after six months when compared to the CG. Improvements in pain were observed after three and six months with the CIG and the SG, with a large effect size after the interventions. Inter-group, both the CIG and SG showed effective improvements after six months when compared to the CG. The foot function and general foot health demonstrated increases after three and six months in both the CIG and SG, with a medium and large effect size. Inter-group, the CIG and SG did not show improvement for foot function and health compared to the CG after three and six months. With respect to the shoe, the CIG and SG showed effectiveness after three and six months, with a large effect size for the CIG and small for the SG. Inter-group, both the CIG and SG showed improvements compared to the CG (Table 3). 

After three and six months with the CIG and SG, the women presented improvements in the six-minute walk test, with a large effect size for the CIG and moderate for the SG. Inter-group, the CIG and SG were different when compared to the CG after three and six months (Table 4).

### 3.2. Secondary Outcome

The biomechanical parameters of plantar pressure distribution after interventions in women with PF revealed that (Table 5): (A) Contact area: forefoot showed a decrease after six months with CIG. Inter-group, a significant reduction was observed in the CIG compared to the SG. In the midfoot, the SG and CIG showed decreases after six months of the intervention. For the medial and lateral rearfoot, only the CIG presented decreases after six months of intervention; (B) Maximum force: the forefoot demonstrated an effective decrease in the CIG after three and six months of intervention. The midfoot showed an effective reduction with both the CIG and SG after six months of intervention. For the medial and lateral rearfoot, the CIG and SG showed effective decreases after three and six months of intervention; (C) Peak plantar pressure: the forefoot and midfoot demonstrated decreases after six months in the SG and CIG. Inter-group, the CIG resulted in a decrease compared to the CG after six months of intervention. For the medial rearfoot, only the CIG demonstrated a decrease after six months of intervention. Inter-group, only the CIG led to a reduction in relation to the SG, but still increased in relation to the CG. For the lateral rearfoot, the SG and CIG led to reductions after six months of treatment. Inter-group, the CIG showed a reduction in relation to the SG, but still increased in relation to the CG.

## 4. Discussion

This was the first clinical trial to compare the therapeutic effectiveness of a custom orthopedic insole in conjunction with a minimalist flexible shoe and the shoe alone. A gait-training protocol with the CIG and SG followed up after three and six months, used for six hours a day, seven days a week (42 h/week), was shown to be effective to improve pain and functionality, and reduce plantar pressure in women with PF, with greater emphasis on the use of the insole (CIG).

The main results showed that the custom insole (CIG) and minimalistic shoe (SG) were beneficial for decreasing foot pain. The CIG was more effective in reducing pain compared to the SG after three and six months. Calcaneus pain during gait and other activities of daily living prompts patients with PF to seek conservative mechanical treatment in combination with other therapies, such as pharmacological and physical therapy [49]. Economic evidence showed that the annual costs of PF are around of USD284 million [1]. This does not include the cost of absences from work that leads to salary reduction, a societal burden, with reduced quality of life, and a psychological burden [1,2,49]. The typical cost per clinical visit is approximately USD50 and the average cost of the use of NSAIDs, a common first-line treatment to reduce foot pain (calcaneus), is nearly USD600 per patient per year [1,2]. Conservative treatments, such as an orthotic customized insole on the calcaneus or plantar arch and night splints on the plantar fascia cost approximately USD500 [1,49,50]. An orthotic insole in conjunction with night splints was shown to be more efficient in reducing calcaneus pain when compared to an insole alone [50]. The literature shows evidence of the beneficial therapeutic effects of the heel insole on pain reduction, in a short period of around 2 to 3 months [24,25,26]. A recent study showed that participants with the custom-made insole, with a short follow-up of 26 weeks, experienced statistically significantly more pain during activity than participants in the usual care (overall effect 1.06; 95% CI 0.36 to 1.75). Total societal costs of the custom-made insole were not significantly higher than those in the usual care group (mean difference EUR376; 95% CI −EUR1775 to EUR2038). The intervention with custom-made insoles was dominated by usual care by the GP (i.e., more expensive and less effective) for pain during activity and quality-of-life outcomes, but for the pain-at-rest outcome, the intervention was more expensive and more effective than usual care. Thus, the cost effectiveness was only 0.59 at very high ceiling ratios [37].

What distinguished this study was to show the greater therapeutic efficacy of the combination of a customized insole with a minimalist flexible shoe, as a low-cost intervention to decrease heel pain in women with PF compared to the shoe alone (cost effectiveness of BRL 20.0 in Brazil, which corresponds to USD 96.0) after the intervention periods of three and six months, considered short and long term. These findings are in agreement with scientific reports on the effectiveness of inexpensive flexible shoes for pain reduction in inflammatory and degenerative diseases, such as knee osteoarthritis [39,40,41,42,43,44].

Foot function improved with both the use of the insole (CIG) and minimalistic shoe (SG) after three and six months, but the CIG showed an effective improvement when compared to the SG and CG after six months of treatment. In the clinical routine, a practical guide from the American College of Foot and Ankle Surgeons recommends non-steroidal anti-inflammatory drugs, stretching, and ankle mobility exercises as the initial steps for the conservative treatment of PF, as well as orthoses and/or insole prescription, as effective conservative secondary treatment options [15,50,51]. The combination between the insole and the minimalist shoe showed great effectiveness for pain relief and improved foot function, a fact that could indicate this intervention to be one of the first steps for conservative treatment, given its low cost, which is an indispensable element, and the fact that the shoe has high clinical relevance for controlling foot support during gait in women with PF. The effectiveness of this foot support can also be explained by the decrease in the Foot Posture Index (FPI) after three and six months of the intervention with the SG and CIG and a reduction in the plantar load. 

According to the literature, the etiology of PF is still unknown; however, it is believed to be multifactorial, with changed biomechanics and delayed healing as likely contributing risk factors [2,3]. A recent review study in 2019 demonstrated that full-length functional insoles are more effective in reducing pain and improving foot function and pronation mechanical control [36]. According to the authors, combining night splints (plantar fascia) or rocker shoes with insoles enhances pain relief and increased foot function compared with rocker shoes and insoles alone [36]. Another study showed that an associated prescription of rocker-soled shoes and custom-made foot insoles led to greater immediate therapeutic effects (pain and foot function) than when each treatment was individually prescribed [52]. The great clinical relevance of this study was to verify the mechanical benefits that the shoe with a minimalist sole, associated with a customized insole, promoted pain relief, improved foot function (medium effect size for the CIG and small for the SG), increased the general health status of the feet, with large effect sizes for the CIG and SG, shoe domain (large effect size for the CIG and small for the SG), and pronation control after six months of the intervention program in women with PF, with the advantage of presenting a low cost for these patients.

Landorf et al. [28], comparing two types of insoles, custom and prefabricated, in patients with PF, showed benefits in functionality and foot health after three months of treatment and 12 months of follow-up. In the current study, the follow-up was for 3 and 6 consecutive months of intervention and comparisons with CG, showing greater effectiveness with the use of the total-contact custom insole (with a wedge at the lateral edges of the calcaneus) after six months of intervention. Another important observation was the increase in the six-minute walk test distance after use of both the insole (CIG) and minimalistic shoe (SG), but with a large effect size for the CIG. Similar findings in improved walking were observed in a clinical trial on the effect of the total-contact insole in individuals with PF after 30, 90, and 180 days [35].

A study using three-dimensional analysis with an infinite element model of the ankle–foot segment revealed that elevated heel height (calcaneus) can significantly change foot biomechanics, especially the strain of force on the anterior talofibular ligament (ATL) and plantar fascia during balance and gait. The authors concluded that the tension force on the plantar fascia decreased at moderate heel height [53]. These findings follow the line of reasoning in the present study, in which the customized insole combined with the shoe with a minimalist flexible sole in the heel resulted in pain relief and improved functionality and foot health, as well as increasing walking distance. Still in this line of reasoning, a recent clinical trial in 2020, carried out on plantar fasciotomy patients, revealed that flip-flop sandals with insoles for 12 weeks were beneficial for decreasing pain and improving foot function [54], agreeing with the present study on women with PF. 

In the current study, the strain force on the ATL increased with the increase in heel height, while the tension force on the plantar fascia decreased at moderate heel height [54]. Patients with unilateral PF and those with bilateral PF showed an increase in the plantar load applied on the rearfoot, midfoot, and forefoot [55]. Our results show the mechanical effectiveness of the intervention with the CIG in relation to the SG to decrease the plantar pressure on the foot, especially the heel. According to the literature, the minimalist shoe can better dissipate plantar loads on the midfoot and forefoot in inflammatory diseases [39,40,41,42,43].

The limitation of this study was that we did not record the plantar fascia thickness by ultrasound over the six months of follow-up with use of the insole and minimalistic shoe (interventions with gait-training protocol); however, our main concern, in this first instance, was to evaluate variables related to pain, function, and calcaneus plantar load in women with PF. Future studies monitoring the plantar fascia thickness may help health professionals to better understand the response strategies to therapeutic mechanical treatment of the disease.

## 5. Conclusions

A customized insole associated with a minimalist flexible shoe during a gait-training protocol can be recommended as a conservative mechanical treatment, which is more effective than the minimalist flexible shoe alone, over the short and long term, for reducing calcaneus pain, increasing function and foot health, and improving walking through reduced plantar load in women with PF.

## Figures and Tables

**Table 1:** Demographic and anthropometric data in the comparisons between women with plantar fasciitis (PF) and control (CG), for the different moments: baseline (T0) and after three (T3) and six months (T6) of intervention with the minimalist shoe (SG) and custom insole in the shoe (CIG).

Outcomes	Intervention (Months)	PF—SG (*n* = 12)	PF—CIG (*n* = 14)	Control—CG (*n* = 10)	p *
Age (years)	T0	46.4 ± 9.6	48.9 ± 9.8	46.1 ± 10.7	0.740
Height (m)	T0	1.60 ± 0.2	1.60 ± 0.1	1.61 ± 0.2	0.144
Body mass (kg)	T0	72.2 ± 10.5	68.2 ± 13.8	70.6 ± 13.6	0.760
	T3	70.0 ± 11.2	66.6 ± 12.1	69.8 ± 11.7	0.708
	T6	71.6 ± 10.5	68.6 ± 12.8	70.8 ± 12.6	0.846
	P	0.894	0.911	0.687	
Body mass index (kg/m^2^)	T0	28.8 ± 4.2	26.7 ± 5.6	26.4 ± 4.8	0.042
	T3	28.5 ± 5.8	26.1 ± 5.1	27.7 ± 5.6	0.498
	T6	29.2 ± 5.3	26.8 ± 4.8	28.2 ± 5.2	0.369
	*p*	0.931	0.958	0.832	

* Two-way ANOVA, considering significant differences *p* < 0.005.

**Table 2:** Pain, function, and posture of the feet in the inter-group comparisons: minimalist shoe (SG), custom insole (CIG), and control group (CG), and interventions: T0 (baseline) and after T3 and T6 months of intervention in women with plantar fasciitis (PF).

Outcomes	Intervention (Gait Training)	T0 (1)	T3 (2)	T6 (3)	Effect Size	95% CI T6	*p*
Foot pain (cm)	SG (1)	7.6 ± 2.0	4.0 ± 1.0	2.5 ± 0.7	0.34	3.8–6.3	0.001 ^1-2; 1-3^ 0.123 ^2-3^
	CIG (2)	7.5 ± 1.7	3.2 ± 0.8	1.5 ± 0.9	0.44	4.9–7.0	0.001 ^1-2;1-3^ 0.013 ^2-3^
	*p*	0.218	0.005 *	0.028 *			
Foot Function Index—(FFI) (0–100)	SG (1)	78.0 ± 16.1	48.9 ± 17.4	22.7 ± 6.4	0.45	44.9–65.6	0.015 ^1-2; 1-3^ 0.035 ^2-3^
	CIG (2)	67.2 ± 18.4	39.6 ± 19.3	18.6 ± 5.3	0.36	38.0–59.1	0.012 ^1-2; 1-3^ 0.009 ^2-3^
	CG (3)	24.2 ± 5.5	25.6 ± 7.3	26.4 ± 8.6	0.30	4.5–8.9	0.903
	*p*	0.012 ^1-3; 2-3^	0.001 ^1-3; 2-3^	0.012 ^1-2; 1-3; 2-3^			
Foot Posture Index (FPI-6) Right (total score)	SG (1)	6.1 ± 4.8	5.6 ± 4.5	5.5 ± 1.7	0.16	2.4–3.6	0.017 ^1-3^
	CIG (2)	6.1 ± 5.1	5.1 ± 4.2	5.2 ± 2.8	0.21	2.2–4.0	0.019 ^1-3^
	CG (3)	3.7 ± 2.2	3.9 ± 2.5	4.0 ± 2.6	0.02	1.9–2.5	0.671
	*p*	0.012 ^1-3; 2-3^	0.001 ^1-3; 2-3^	0.012 ^1-3; 2-3^			
Foot Posture Index (FPI-6) Left (total score)	SG (1)	6.2 ± 4.9	5.7 ± 4.6	5.5 ± 2.1	0.18	2.4–3.9	0.001 ^1-3^
	CIG (2)	6.0 ± 5.1	5.0 ± 4.8	5.0 ± 2.5	0.24	2.1–4.2	0.027 ^1-3^
	CG (3)	4.7 ± 3.6	4.3 ± 3.0	4.8 ± 2.9	0.03	2.9–3.1	0.664
	*p*	0.012 ^1-3; 2-3^	0.003 ^1-3; 2-3^	0.004 ^1-3; 2-3^			

* Two-way ANOVA, interventions: baseline T0 (1) and after T3 (2) and T6 (3) months, and groups (SG, CIG, and CG), followed by Tukey’s post hoc test, significant differences *p* < 0.005 and 95% CI (T6). Effect size with Cohen’s D between T0 and after six (T6) months of intervention.

**Table 3:** Foot Health Status and its domains in the inter-group comparisons: minimalist shoe (SG), custom insole (CIG), and control group (CG), and interventions: T0 (baseline) and after T3 and T6 months of intervention in women with plantar fasciitis (PF).

Outcomes	Intervention (Gait Training)	T0 (1)	T3 (2)	T6 (3)	Effect Size	95% CI T6	*p* *
Foot Health Status—(FSHQ-Br) (0–100)	SG (1)	48.8 ± 11.0	59.6 ± 14.1	90.7 ± 9.5	0.35	33.2–50.6	0.010 ^1-3^ 0.024 ^2-3^
	CIG (2)	48.3 ± 17.0	60.1 ± 16.7	94.5 ± 6.9	0.40	36.1–56.3	0.002 ^1-3^ 0.001 ^2-3^
	CG (3)	84.4 ± 7.9	85.1 ± 6.7	85.4 ± 6.9	0.05	33.2–50.6	0.345
	*p*	0.011 ^1-3; 2-3^	0.002 ^1-3; 2-3^	0.166			
Foot Pain	SG (1)	5.3 ± 1.5	8.8 ± 2.4	16.2 ± 2.3	0.81	9.2–12.5	0.011 ^1-2; 1-3^ 0.289 ^2-3^
	CIG (2)	3.3 ± 1.5	5.3 ± 2.0	17.4 ± 3.6	0.88	11.9–16.2	0.010 ^1-2; 1-3^ 0.072 ^2-3^
	CG (3)	17.8 ± 2.6	17.6 ± 2.5	17.9 ± 2.9	0.03	2.4–2.6	0.128
	*p*	0.006 ^1-3; 2-3^	0.003 ^1-3;2-2^	0.358			
Foot Function	SG (1)	3.4± 1.7	4.8 ± 2.3	17.5 ± 4.5	0.41	11.2–16.9	0.010 ^1-2; 1-3^ 0.035 ^2-3^
	CIG (2)	3.0 ± 1.0	5.7 ± 2.6	20.1 ± 4.0	0.58	14.8–19.3	0.005 ^1-2; 1-3^ 0.040 ^2-3^
	CG (3)	25.6 ± 1.7	25.0 ± 1.8	25.2 ± 1.6	0.14	1.1–1.9	0.423
	*p*	0.003 ^1-3; 2-3^	0.001 ^1-3; 2-3^	0.001 ^1-3; 2-3^			
Footwear	SG (1)	6.5 ± 2.8	7.1 ± 4.9	7.2 ± 4.5	0.25	2.4–3.8	0.001 ^1-2; 1-3^ 0.922 ^2-3^
	CIG (2)	4.2 ± 1.0	6.3 ± 3.1	8.1 ± 4.2	0.81	1.5–6.2	0.011 ^1-2; 1-3^ 0.019 ^2-3^
	CG (3)	9.6 ± 3.6	10.0 ± 4.0	10.5 ± 4.7	0.21	3.0–4.8	0.254
	*p*	0.001 ^1-3; 2-3^	0.179	0.488			
General Foot Health	SG (1)	3.3 ± 2.4	4.3 ± 2.1	7.5 ± 2.6	0.80	2.0–6.3	0.003 ^1-2; 1-3^ 0.424 ^2-3^
	CIG (2)	3.1 ± 2.1	4.0 ± 2.7	8.6 ± 1.8	0.83	3.9–7.0	0.001 ^1-2; 1-3^ 0.469 ^2-3^
	CG (3)	13.8 ± 3.8	13.9 ± 3.7	14.2 ± 4.2	0.09	3.3–4.1	0.233
	*p*	0.001 ^1-3; 2-3^	0.001 ^1-3; 2-3^	0.001 ^1-3; 2-3^			

* Two-way ANOVA, interventions: baseline T0 (1) and after T3 (2) and T6 (3) months, and groups (SG, CIG, and CG), followed by Tukey’s post hoc test, significant differences *p* < 0.005 and 95% CI (T6). Effect size with Cohen’s D between T0 and after six (T6) months of intervention.

**Table 4:** Six-minute walk test (6MWT) in the inter-group comparisons: minimalist shoe (SG), custom insole (CIG), and control group (CG), and interventions: T0 (baseline) and after T3 and T6 months of intervention in women with plantar fasciitis (PF).

6MWT	Intervention	T0 (1)	T3 (2)	T6 (3)	Effect size	95% CI T6	*p* *
Walking distance (m)	SG (1)	387.5 ± 99.0	535.8 ± 59.0	556.0 ± 94.6	0.74	86.5–250.4	0.001 ^1−2; 1−3^ 0.735 ^2−3^
	CIG (2)	385.0 ± 75.5	540.8 ± 35.3	584.2 ± 41.2	0.82	140.3–258.1	0.010 ^T1−2; 1−3^ 0.795 ^2−3^
	CG (3)	741.2 ± 56.0	723.2 ± 52.9	742.8 ± 54.7	0.03	73.5–76.7	0.734
	*p*	<0.001 ^1−3; 2−3^	0.004 ^1−3; 2−3^	0.013 ^1−3; 2−3^			
Number of turns (*n*)	SG (1)	13.3 ± 4.4	18.8 ± 3.5	19.3 ± 4.2	0.79	2.3–9.6	0.002 ^1−2; 1−3^ 0.185 ^2−3^
	CIG (2)	13.1 ± 2.6	17.7 ± 1.2	20.6 ± 2.0	0.88	5.6–9.3	0.001 ^T1−2; 1−3^ 0.164 ^2−3^
	CG (3)	24.0 ± 1.6	23.0 ± 1.5	24.0 ± 1.4	0.02	1.3–1.4	0.568
	*p*	<0.001 ^1−3; 2−3^	0.026 ^1−3; 2−3^	<0.001 ^1−3; 2−3^			

* Two-way ANOVA, interventions: baseline T0 (1) and after T3 (2) and T6 (3) months, and groups (SG, CIG, and CG), followed by Tukey’s post hoc test, significant differences *p* < 0.005 and 95% CI (T6). Effect size with Cohen’s D between T0 and after six (T6) months of intervention.

**Table 5:** Plantar pressure distribution in the inter-group comparisons: minimalist shoe (SG), custom insole (CIG), and control group (CG), and interventions: T0 (baseline) and after T3 and T6 months of intervention of the women with plantar fasciitis (PF).

Plantar Pressure	Intervention (Gait Training)	T0 (1)	T3 (2)	T6 (3)	Effect size	95% CI T6	*p* *
Forefoot Contact area (cm)	SG (1) CIG (2)	15.2 ± 7.9 14.0 ± 5.0	12.3 ± 1.5 10.9 ± 1.4	13.6 ± 3.2 13.4 ± 3.6	0.26 0.23	3.5–6.7 2.7–3.9	0.134 0.001 ^1-3; 2-3^
	CG (3)	11.5 ± 1.7	11.8 ± 1.5	12.0 ± 1.3	0.24	1.0–1.9	0.483
	*p*	0.001 ^1-3; 2-3^	0.044 ^1-2^	0.162			
Midfoot Contact area (cm)	SG (1) CIG (2)	30.2 ± 7.3 27.9 ± 10.8	29.9 ± 9.2 20.8 ± 8.5	24.3 ± 7.8 18.9 ± 7.2	0.78 0.98	1.5–12.2 1.8–16.1	0.025 ^1-3^ 0.001 ^1-2; 1-3^
	CG (3)	24.1 ± 11.4	23.9 ± 8.9	24.6 ± 9.2	0.04	9.2–10.2	0.989
	*p*	0.046 ^1-3; 2-3^	0.082	0.192			
Medial Rearfoot Contact area (cm)	SG (1) CIG (2)	21.1 ± 2.2 20.5 ± 2.9	22.7 ± 3.2 19.9 ± 3.0	21.2 ± 3.0 18.9 ± 3.7	0.13 0.48	2.1–2.3 1.0–4.1	0.167 0.001 ^1-3^
	CG (3)	20.3 ± 3.5	21.4± 5.4	20.9 ± 4.5	0.14	3.1–4.3	0.951
	*p*	0.453	0.034 ^2-3^	0.605			
Lateral Rearfoot Contact area (cm)	SG (1) CIG (2)	22.3 ± 3.3 21.1 ± 4.4	24.5 ± 3.9 20.7 ± 2.5	22.6 ± 2.4 20.6 ± 2.0	0.10 0.14	2.1–2.7 2.2–3.1	0.173 0.006 ^1-3^
	CG (3)	20.6 ± 3.9	21.2 ± 3.3	21.8 ± 3.8	0.19	2.4–4.8	0.853
	*p*	0.521	0.018 ^2-3^	0.195			
Forefoot Maximum force (N/BW)	SG (1) CIG (2)	0.18 ± 0.03 0.18 ± 0.02	0.18 ± 0.02 0.17 ± 0.03	0.17 ± 0.03 0.16 ± 0.02	0.33 0.80	0.01–0.03 0.02–0.03	0.564 0.001 ^1-2; 1-3^
	CG (3)	0.18 ± 0.03	0.17 ± 0.05	0.18 ± 0.07	0.05	0.05–0.06	0.789
	*p*	0.842	0.208	0.113			
Midfoot Maximum force (N/BW)	SG (1) CIG (2)	0.24 ± 0.11 0.20 ± 0.14	0.24 ± 0.13 0.15 ± 0.08	0.15 ± 0.09 0.13 ± 0.08	0.89 0.61	0.04–0.17 0.02–0.15	0.023 ^1-3^ 0.029 ^1-3^
	CG (3)	0.15 ± 0.05	0.15 ± 0.03	0.16 ± 0.04	0.22	0.03–0.05	0.886
	*p*	0.001 ^1-3; 2-3^	0.068	0.682			
Medial rearfoot Maximum force (N/BW)	SG (1) CIG (2)	0.37 ± 0.06 0.37 ± 0.06	0.41 ± 0.09 0.35 ± 0.08	0.35 ± 0.09 0.33 ± 0.07	0.26 0.61	0.04–0.08 0.02–0.10	0.001 ^1-2; 1-3^ 0.110 ^2-3^ 0.001 ^1-2; 1-3^ 0.264 ^2-3^
	CG (3)	0.31 ± 0.09	0.30 ± 0.06	0.30 ± 0.07	0.12	0.06–0.08	0.699
	*p*	0.001 ^1-3; 2-3^	0.010 ^1-3; 2-3^	0.425			
Lateral rearfoot Maximum force (N/BW)	SG (1) CIG (2)	0.38 ± 0.06 0.37 ± 0.09	0.44 ± 0.04 0.35 ± 0.06	0.37 ± 0.08 0.34 ± 0.07	0.15 0.37	0.04–0.08 0.02–0.15	0.005 ^1-2; 1-3^ 0.239 ^2-3^ 0.001 ^1-2; 1-3^ 0.112 ^2-3^
	CG (3)	0.31 ± 0.05	0.31 ± 0.07	0.31 ± 0.06	0.03	0.03–0.05	0.831
	*p*	0.001 ^1-3; 2-3^	0.001 ^1-3; 2-3^	0.173			
Forefoot Peak Pressure (kPa)	SG (1) CIG (2)	323.5 ± 46.8 304.0 ± 30.7	312.7 ± 41.2 299.5 ± 29.7	294.0 ± 43.0 274.5 ± 39.4	0.65 0.83	8.5–67.5 2.0–56.9	0.048 ^1-3^ 0.013 ^1-3^ 0.125 ^2-3^
	CG (3)	295.1 ± 25.0	294.2 ± 26.7	296.4 ± 26.3	0.05	22.8–25.4	0.774
	*p*	0.001 ^1-3; 2-3^	0.436	0.008 ^1-2; 2-3^			
Midfoot Peak Pressure (kPa)	SG (1) CIG (2)	176.6 ± 23.3 176.4 ± 44.8	173.1 ± 40.3 164.8 ± 41.9	154.0 ± 35.1 149.9 ± 47.7	0.75 0.57	2.6–47.8 9.4–62.4	0.002 ^1-3^ 0.149 ^2-3^ 0.010 ^1-3^ 0.133 ^2-3^
	CG (3)	153.3 ± 37.6	153.9 ± 32.6	155.4 ± 37.5	0.05	33.1–37.3	0.688
	*p*	0.024 ^1-3; 2-3^	0.455	0.503			
Medial rearfoot Peak Pressure (kPa)	SG (1) CIG (2)	310.6 ± 40.0 317.9 ± 56.5	318.4 ± 44.8 308.1 ± 49.0	309.5 ± 56.3 303.4 ± 27.0	0.02 0.32	40.2–42.4 20.0–48.9	0.563 0.001 ^1-3^ 0.632 ^2-3^
	CG (3)	292.7 ± 47.0	290.3 ± 42.6	287.8 ± 40.3	0.22	36.2–46.0	0.876
	*p*	0.007 ^1-3; 2-3^	0.317	0.012 ^1-2; 2-3^			
Lateral rearfoot Peak Pressure (kPa)	SG (1) CIG (2)	292.3 ± 38.8 311.9 ± 57.4	317.7 ± 41.9 298.1 ± 43.2	310.4 ± 54.7 295.9 ± 23.7	0.38 0.36	22.0–58.2 18.1–50.1	0.002 ^1-3^ 0.236 ^2-3^ 0.001 ^1-3^ 0.530 ^2-3^
	CG (3)	289.5 ± 51.2	286.2 ± 57.3	283.5 ± 55.9	0.11	44.3–56.3	0.982
	*p*	0.025 ^1-3; 2-3^	0.394	0.012 ^1-2; 2-3^			

* Two-way ANOVA, interventions: baseline T0 (1) and after T3 (2) and T6 (3) months, and group (SG, CIG, and CG), followed by Tukey’s post hoc test, significant differences *p* < 0.005 and 95% CI (T6). Effect size with Cohen’s D between T0 and after six (T6) months of intervention.

## Data Availability

The datasets generated and/or analyzed during the current study are not publicly available due to limitations of ethical approval involving the patient data and anonymity, but are available from the corresponding author (apribeiro@alumni.usp.br) on reasonable request.

## References

[1] Tong K.B., Furia J. (2010). Economic burden of plantar fasciitis treatment in the United States. Am. J. Orthop..

[2] Thomas M.J., Whittle R., Menz H.B., Rathod-Mistry T., Marshall M., Roddy E. (2019). Plantar heel pain in middle-aged and older adults: Population prevalence, associations with health status and lifestyle factors, and frequency of healthcare use. BMC Musculoskelet. Disord..

[3] Ribeiro A.P., João S.M.A., Dinato R.C., Tessutti V.D., Sacco I.C.N. (2015). Dynamic Patterns of Forces and Loading Rate in Runners with Unilateral Plantar Fasciitis: A Cross-Sectional Study. PLoS ONE.

[4] Lemont H., Ammirati K.M., Usen N. (2003). A Degenerative Process (Fasciosis) Without Inflammation. JAPMA.

[5] Young C.C., Rutherford D.S., Niedfeldt M.W. (2001). Treatment of plantar fasciitis. Am. Fam. Phys..

[6] Bedi H.S., Love B.R. (1998). Differences in impulse distribution in patients with plantar fasciitis. Foot Ankle Int..

[7] Wearing S.C., Smeathers J.E., Urry S.R. (2003). The effect of plantar fasciitis on vertical foot-ground reaction force. Clin. Orthop. Relat. Res..

[8] Wearing S.C., Smeathers J.E., Sullivan P.M., Yates B., Urry S.R., Dubois P. (2007). Plantar fasciitis: Are pain and fascial thickness associated with arch shape and loading?. Phys Ther..

[9] Pavan P.G., Stecco C., Darwish S., Natali A.N., De Caro R. (2011). Investigation of the mechanical properties of the plantar aponeu-rosis. Surg. Radiol. Anat..

[10] Schepsis A.A., Leach R.E., Gorzyca J. (1991). Plantar fasciitis. Etiology, treatment, surgical results, and review of the literature. Clin. Orthop. Relat. Res..

[11] League A.C. (2008). Current Concepts Review: Plantar Fasciitis. Foot Ankle Int..

[12] Taunton J.E., Ryan M.B., Clement D.B., McKenzie D.C., Lloyd-Smith D. (2002). Plantar fasciitis: A retrospective analysis of 267 cases. Phys. Ther. Sport.

[13] Taunton E.J., Ryan M.B., Clement D.B., McKenzie D.C., Lloyd-Smith D.R., Zumbo B.D. (2002). A retrospective case-control analysis of 2002 running injuries. Br. J. Sports Med..

[14] Pohl M.B., Hamil J., David I.S. (2009). Biomechanical and anatomic factors associated with a history of plantar fasciitis in female runners. Clin. J. Sport Med..

[15] Thomas J.L., Christensen J.C., Kravitz S.R., Mendicino R.W., Schuberth J.M., Vanore J.V., Baker J. (2010). American College of Foot and Ankle Surgeons heel pain committee. The diagnosis and treatment of heel pain: A clinical practice guideline-revision. J. Foot Ankle Surg..

[16] Schwartz E.N., Su J. (2014). Plantar Fasciitis: A Concise Review. Perm. J..

[17] Khan M.N., Jacobs B.C., Ashbaugh S. (2013). Considerations in Footwear and Orthotics. Prim. Care: Clin. Off. Pr..

[18] López A.M.D., Carrasco P.G. (2014). Effectiveness of Different Physical Therapy in Conservative Treatment of Plantar Fasciitis. Systematic Review. Rev. Esp. Salud Pública.

[19] Young C. (2012). In the clinic: Plantar fasciitis. Ann. Intern. Med..

[20] Donley B.G., Moore T., Sferra J., Gozdanovic J., Smith R. (2007). The Efficacy of Oral Nonsteroidal Anti-Inflammatory Medication (NSAID) in the Treatment of Plantar Fasciitis: A Randomized, Prospective, Placebo-Controlled Study. Foot Ankle Int..

[21] Peerbooms J.C., van Laar W., Faber F., Schuller H.M., van der Hoeven H., Gosens T. (2010). Use of platelet rich plasma to treat plantar fasciitis: Design of a multi centre randomized controlled trial. BMC Musculoskelet. Disord..

[22] Li Z., Xia C., Yu A., Qi B. (2014). Ultrasound- versus Palpation-Guided Injection of Corticosteroid for Plantar Fasciitis: A Meta-Analysis. PLoS ONE.

[23] Santa-Coloma E., Khoury M.A. (2011). Medicina basada enla evidencia: Evidencia enelmanejo no quirúrgico dela fascitis plantar. Rev. Asoc. Argent Traumatol. Deporte..

[24] Lynch D.M., Goforth W.P., Martin E.J., Odom R.D., Preece C.K., Kotter M.W. (1998). Conservative treatment of plantar fasciitis. A prospective study. J. Am. Podiatr. Med. Assoc..

[25] Pfeffer G., Bacchetti P., Deland J., Lewis A., Anderson R., Davis W., Alvarez R., Brodsky J., Cooper P., Frey C. (1999). Comparison of Custom and Prefabricated Orthoses in the Initial Treatment of Proximal Plantar Fasciitis. Foot Ankle Int..

[26] Martin J.E., Hosch J.C., Goforth W.P., Murff R.T., Lynch D.M., Odom R.D. (2001). Mechanical Treatment of Plantar Fasciitis: A prospective study. J. Am. Podiatr. Med Assoc..

[27] Roos E., Engström M., Söderberg B. (2006). Foot Orthoses for the Treatment of Plantar Fasciitis. Foot Ankle Int..

[28] Landorf K.B., Keenan A.M., Herbert R.D. (2006). Effectiveness of foot orthoses to treatment plantar fasciitis: A randomized trial. Arch. Intern Med..

[29] DiGiovanni B.F., Nawoczenski D.A., Malay D.P., Graci P.A., Williams T.T., Wilding G.E., Baumhauer J.F. (2006). Plantar fasica-specific stretching exercise improves outcomes in patients with chronic plantar fasciitis. A prospective clinical trial with two-year follow-up. J. Bone Joint Surg. Am..

[30] Lee Y.-C., Lin G., Wang M.-J.J. (2012). Evaluating insole design with joint motion, plantar pressure and rating of perceived exertion measures. Work.

[31] Antwi-Afari M.F., Li H., Yu Y., Kong L. (2018). Wearable insole pressure system for automated detection and classification of awkward working postures in construction workers. Autom. Constr..

[32] Curuk E., Aruin A.S. (2021). The effect of a textured insole on anticipatory postural adjustments. Somatosens. Mot. Res..

[33] Pinvanichkul C., Siriphorn A. (2021). Effect of Walking Training with Textured Insole Socks in Older Adults. Phys. Occup. Ther. Geriatr..

[34] Haris F., Liau B.-Y., Jan Y.-K., Akbari V.B.H., Primanda Y., Lin K.-H., Lung C.-W. (2021). A Review of the Plantar Pressure Distribution Effects from Insole Materials and at Different Walking Speeds. Appl. Sci..

[35] Oliveira H.A.V., Jones A., Moreira E., Jennings F., Natour J. (2015). Effectiveness of Total Contact Insoles in Patients with Plantar Fasciitis. J. Rheumatol..

[36] Schuitema D., Greve C., Postema K., Dekker R., Hijmans J.M. (2020). Effectiveness of Mechanical Treatment for Plantar Fasciitis: A Systematic Review. J. Sport Rehabil..

[37] Rasenberg N., Bierma-Zeinstra S.M.A., Fuit L., Rathleff M.S., Dieker A., van Veldhoven P., Bindels P.J.E., van Middelkoop M. (2021). Custom insoles versus sham and GP-led usual care in patients with plantar heel pain: Results of the STAP-study—A ran-domised controlled trial. Br. J. Sports Med..

[38] Coheña-Jiménez M., Pabón-Carrasco M., Pérez Belloso A.J. (2021). Comparison between customised foot orthoses and insole com-bined with the use of extracorporeal shock wave therapy in plantar fasciitis, mediumterm follow-up results: A randomised controlled trial. Clin. Rehabil..

[39] Shakoor N., Block J.A. (2006). Walking barefoot decreases loading on the lower extremity joints in knee osteoarthritis. Arthritis Care Res..

[40] Shakoor N., Lidtke R.H., Sengupta M., Fogg L.F., Block J. (2008). Effects of specialized footwear on joint loads in osteoarthritis of the knee. Arthritis Care Res..

[41] Shakoor N., Lidtke R.H., Wimmer M.A., Mikolaitis R.A., Foucher K.C., Thorp L.E., Fogg L.F., Block J.A. (2010). Improvement in Knee Loading After Use of Specialized Footwear for Knee Osteoarthritis: Results of a Six-Month Pilot Investigation. Arthritis Care Res..

[42] Shakoor N., Sengupta M., Foucher K., Wimmer M.A., Fogg L.F., Block J. (2010). Effects of common footwear on joint loading in osteoarthritis of the knee. Arthritis Care Res..

[43] Trombini-Souza F., Kimura A., Ribeiro A.P., Butugan M., Akashi P., Pássaro A.C., Arnone A.C., Sacco I.C. (2011). Inexpensive footwear decreases joint loading in elderly women with knee osteoarthritis. Gait Posture.

[44] Ribeiro A.P., de Souza B.L., João S.M.A. (2022). Effectiveness of mechanical treatment with customized insole and minimalist flexible footwear for women with calcaneal spur: Randomized controlled trial. BMC Musculoskelet. Disord..

[45] Oliveira L.M., Alves C.M., Mizuzaki J., Natour J. (2015). Translation and cross-cultural adaptation of FFI to Brazilian Portuguese version: FFI–Brazil. Rev. Bras. Reumatol..

[46] Ferreira A.F.B., Laurindo I.M.M., Rodrigues P.T., Ferraz M.B., Kowalski S.C., Tanaka C. (2008). Brazilian Version of the Foot Health Status Questionnaire (FHSQ-Br): Cross-Cultural Adaptation and Evaluation of Measurement Properties. Clinics.

[47] Hamilton D.M., Haennel R.G. (2000). Validity and Reliability of the 6-Minute Walk Test in a Cardiac Rehabilitation Population. J. Cardiopulm. Rehabilitation.

[48] Redmond A.C., Crane Y.Z., Menz H.B. (2008). Normative values for the Foot Posture Index. J. Foot Ankle Res..

[49] Luffy L., Grosel J., Thomas R., So E. (2018). Plantar fasciitis: A review of treatments. JAAPA.

[50] Lee W.C., Wong W.Y., Kung E., Leung A.K. (2012). Effectiveness of adjustable dorsiflexion night splint in combination with accom-modative foot orthosis on plantar fasciitis. J. Rehabil Res. Dev..

[51] Lee S.Y., McKeon P., Hertel J. (2009). Does the use of orthoses improve self-reported pain and function measures in patients with plantar fasciitis? A meta-analysis. Phys. Ther. Sport.

[52] Fong D.T., Pang K.Y., Chung M.M., Hung A.S., Chan K.M. (2012). Evaluation of combined prescription of rocker sole shoes and cus-tom-made foot orthoses for the treatment of plantar fasciitis. Clin. Biomech..

[53] Yu J., Wong D.W.-C., Zhang H., Luo Z.-P., Zhang M. (2016). The influence of high-heeled shoes on strain and tension force of the anterior talofibular ligament and plantar fascia during balanced standing and walking. Med. Eng. Phys..

[54] Costa A.R.A., Silva H.J.D.A., Mendes A.A.M.T., Silva R.S., Lins C.A.D.A., De Souza M.C. (2020). Effects of insoles adapted in flip-flop sandals in people with plantar fasciopathy: A randomized, double-blind clinical, controlled study. Clin. Rehabil..

[55] Yoo S.D., Kim H.S., Lee J.H., Yun D.H., Kim D.H., Chon J., Lee S.A., Han Y.J., Soh Y.S., Kim Y. (2017). Biomechanical Parameters in Plantar Fasciitis Measured by Gait Analysis System With Pressure Sensor. Ann. Rehabil. Med..

